# Swept source optical coherence tomography and optical coherence tomography angiography in pediatric enhanced S-cone syndrome: a case report

**DOI:** 10.1186/s13256-018-1819-4

**Published:** 2018-10-03

**Authors:** Angelo Maria Minnella, Valeria Pagliei, Maria Cristina Savastano, Matteo Federici, Matteo Bertelli, Paolo Enrico Maltese, Giorgio Placidi, Giovanni Corbo, Benedetto Falsini, Aldo Caporossi

**Affiliations:** 1 Institute of Ophthalmology, Università Cattolica del Sacro Cuore – Fondazione Policlinico Universitario A. Gemelli-IRCCS, Rome, Italy; 2 MAGI, Human Medical Genetics Institute, Bolzano, Italy; 3 Department of Ophthalmology, Università La Sapienza, Rome, Italy

**Keywords:** Bilateral schisis, Enhanced S-cone syndrome, “En face” OCT, Hereditary retinal dystrophy, Innovative biotechnology, OCT angiography, S-cone specific ERG, Swept source OCT

## Abstract

**Background:**

Enhanced S-cone syndrome is an autosomal recessive retinal dystrophy related to a defect in a nuclear receptor gene (*NR2E3*) that leads to alteration in cells development from rod to S-cone. This retinal dystrophy may be associated with retinal schisis. The aim of this report is to describe structural optical coherence tomography and optical coherence tomography angiography features in a case of enhanced S-cone syndrome associated with macular schisis.

**Case presentation:**

A Caucasian 13-year-old girl underwent measurement of best corrected visual acuity, ophthalmoscopic evaluation, and fundus autofluorescence examination. Photopic and scotopic electroretinography were carried out as well. Enhanced S-cone syndrome was suspected on the basis of clinical and electrophysiological findings. Structural optical coherence tomography and optical coherence tomography angiography allowed the further characterization of the associated macular schisis.

Genetic analysis not only confirmed the diagnosis but increased the clinical novelty of this case report by showing two variations in the *NR2E3* gene probably related to the phenotype: a missense variation c.1118T>C which leads to the substitution of leucine with proline in amino acid position 373, and c.349+5G>C, which involves a gene sequence near a splicing site.

**Conclusions:**

Swept source structural optical coherence tomography (B scans and “en face” images) and optical coherence tomography angiography allowed the observation of retinal structural details and the involvement of each retinal layer and capillary plexus in enhanced S-cone syndrome. Of interest, neither of the two *NR2E3* gene variants found in this case report have been linked to any form of retinopathy.

## Background

Enhanced S-cone syndrome (ESCS) is an autosomal recessive retinal dystrophy which was first described in 1990 as clinically characterized by night blindness and a nummular pigmentary deposit in the retinal pigment epithelium outside the vascular arcades [1]. These features could also be associated with foveal schisis, resulting in a mild visual acuity loss and visual field loss [2–4].

Genetically, ESCS is related to a defect in the nuclear receptor subfamily 2 group E member 3 gene (*NR2E3*) which encodes a rod-specific ligand-dependent transcription factor that plays a key role in the differentiation of post-mitotic photoreceptor precursor cells [5–8]. Genetic variations in *NR2E3* lead to a loss of function in the transcription factor altering the development of cells from rod to S-cone thus explaining the histopathologic and immunocytochemical features [6]. Analysis of a postmortem eye showed a degenerative retina with no rods and an abnormal increase in the number of cones, mostly S-cones [9]. These retinal findings are consistent with the peculiar response on electroretinography (ERG) which reveals enhanced short-wavelength sensitivity and absent rod function. In addition, a 30 Hz flicker is markedly delayed with an amplitude lower than normal and a cone b-wave loss greater than that of the photopic a-wave [2].

In the literature, the clinical spectrum of ESCS has been previously reported [10] and macular schisis has been described as the appearance of hyporeflective cystic-like spaces and a lack of well-defined and hyporeflective bands in the inner and outer nuclear layer which can be observed by performing optical coherence tomography (OCT). Splitting in the retinal thickness at the level of the outer plexiform layer can be detected as well [4].

OCT angiography (OCT-A), enabling a non-invasive and fast visualization of both retinal capillary plexus and choriocapillaris, has been investigated in hereditary retinal dystrophies such as Stargardt disease [11] and retinitis pigmentosa [12] in order to further understand their pathogenesis. In eyes affected by X-linked retinoschisis, structural OCT has already been used to describe retinal features [13, 14], and OCT-A revealed retinal abnormalities mainly observed at the level of the deep capillary plexus where non-reflective spaces and telangiectasia-like alterations were located [15], as well as accentuated vascular tortuosity, microvascular protrusions, and an enlarged foveal avascular zone [16]; the involvement of the superficial vascular plexus was less evident and the involvement of the choriocapillaris almost absent [15–17].

However, the importance of swept source OCT “en face” images, as well as OCT-A, in characterizing retinoschisis related to ESCS has never been emphasized. The aim of this report is to describe swept source “en face” OCT and OCT-A details in a case of ESCS associated with macular schisis.

## Case presentation

We describe the case of a Caucasian 13-year-old girl who was first referred to our department with a diagnosis of retinitis pigmentosa.

She underwent a full clinical examination which included visual acuity, ophthalmoscopic evaluation, structural OCT, and photopic and scotopic ERG. The autofluorescence of both eyes showed a hyperautofluorescent halo around the fovea covering 360°. According to Gelman *et al.* [18], the “hyperautofluorescent ring border” corresponds to impending photoreceptor loss.

**Fig. 1:**
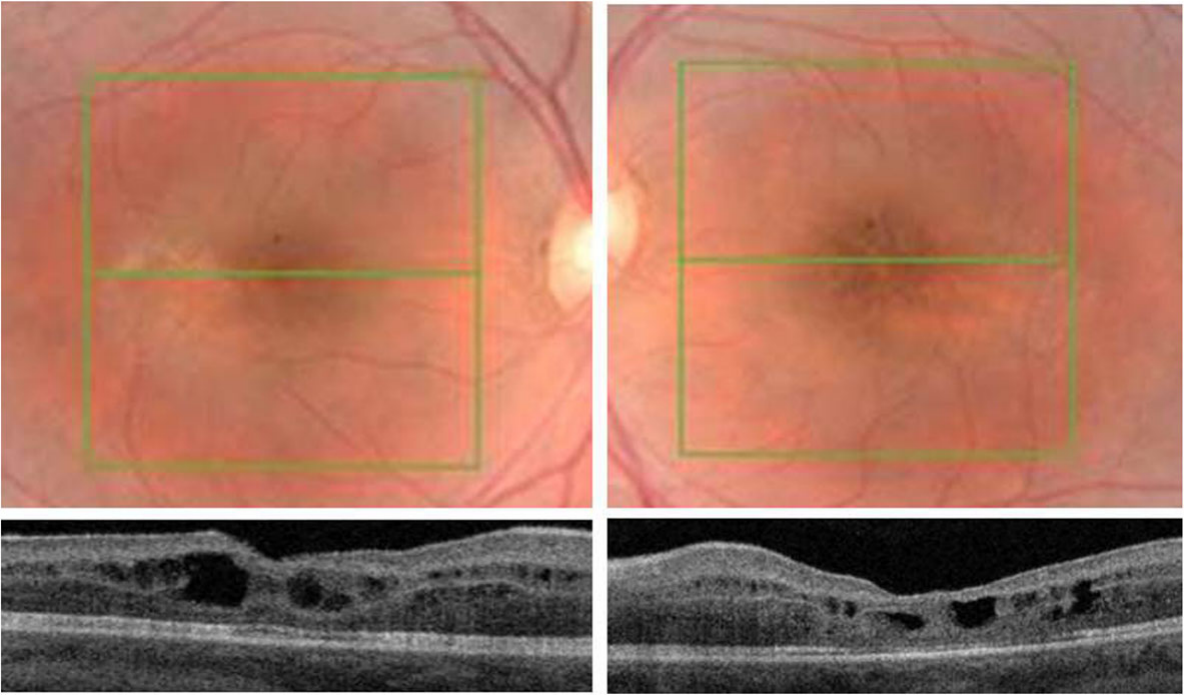
Fundus photography and structural optical coherence tomography B scan of eyes affected by enhanced S-cone syndrome. Macular optical coherence tomography shows a deformation of the retinal profile and the presence of schisis involving the inner and outer nuclear layers. These optical coherence tomography features can be seen in both eyes

Her best corrected visual acuity (BCVA) was 75 letters Early Treatment Diabetic Retinopathy Study (ETDRS) in her right eye (RE) and 60 letters in her left eye (LE). The visual acuity impairment was consistent with the abnormalities detected using OCT which revealed a deformation of the retinal profile and the presence of cystic spaces in both eyes (Fig. 1). This report was referred to as “schisis at an early stage.”

**Fig. 2:**
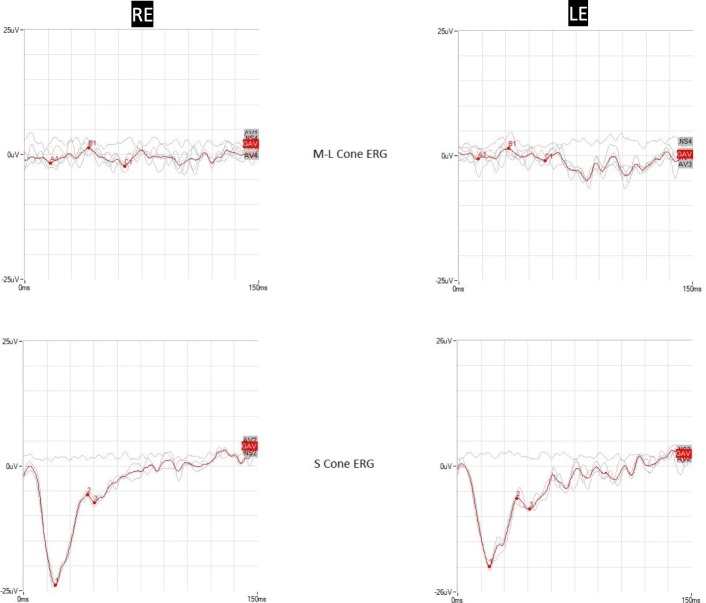
Cone-mediated electroretinograms of the patient affected by enhanced S-cone syndrome. *Top*: M-L cone responses to ganzfeld red stimuli presented on a blue background. *Bottom*: S-cone responses to ganzfeld blue stimuli presented on a yellow background. Note that S-cone responses are abnormally larger in amplitude than M-L cone responses. *ERG* electroretinogram, *LE* left eye, *RE* right eye

ERG, performed according to International Society for Clinical Electrophysiology of Vision (ISCEV) standard, showed non-recordable rod response, and reduced maximal and cone responses with a decreased b-wave to a-wave ratio. Responses to 30 Hz flicker were reduced and delayed. Specialized ERG recordings of responses mediated prevalently by short-wavelength-sensitive cones (S-cones) and ML-wavelength sensitive cones (ML-cones) were obtained from both eyes. S-cone-mediated ERGs were recorded in response to a blue (420 nm) stimulus of 30 degrees flickered at 4 Hz and presented on a steady yellow background. ML-cone-mediated ERGs were obtained in response to a red (580 nm) stimulus of 30 degrees flickering at 4 Hz and presented on a steady blue (420 nm) background. Blue and red stimuli were photopically matched. In normal individuals the ML cone ERG is three times larger in amplitude (a-b wave peak) and 10 ms shorter (b-wave peak) compared to S-cone ERG. In this particular patient, S-cone ERG was of larger amplitude (1.2 times) compared to ML cone. The peak times of both responses were comparable. These findings indicated the enhanced S-cone sensitivity for this patient (Fig. 2).

Highly suspected on the basis of clinical and electrophysiological findings the diagnosis of ESCS was made.

Our patient was evaluated every 6 months for a year. BCVA was measured every time: 6 months after the first examination, it was found to be 65 letters ETDRS in her RE and 75 letters in her LE, while, a year later, it was 82 letters in her RE and 75 letters in her LE.

Two years after the first examination, BCVA decreased to 65 letters in her RE and 40 letters in her LE, consistent with OCT findings. Structural OCT was performed using the DRI OCT Triton™ swept source OCT device (Topcon Medical Tokyo, Japan) and revealed an increased retinal thickness and a markedly altered retinal structure due to macular schisis. These features were detected in both eyes.

**Fig. 3:**
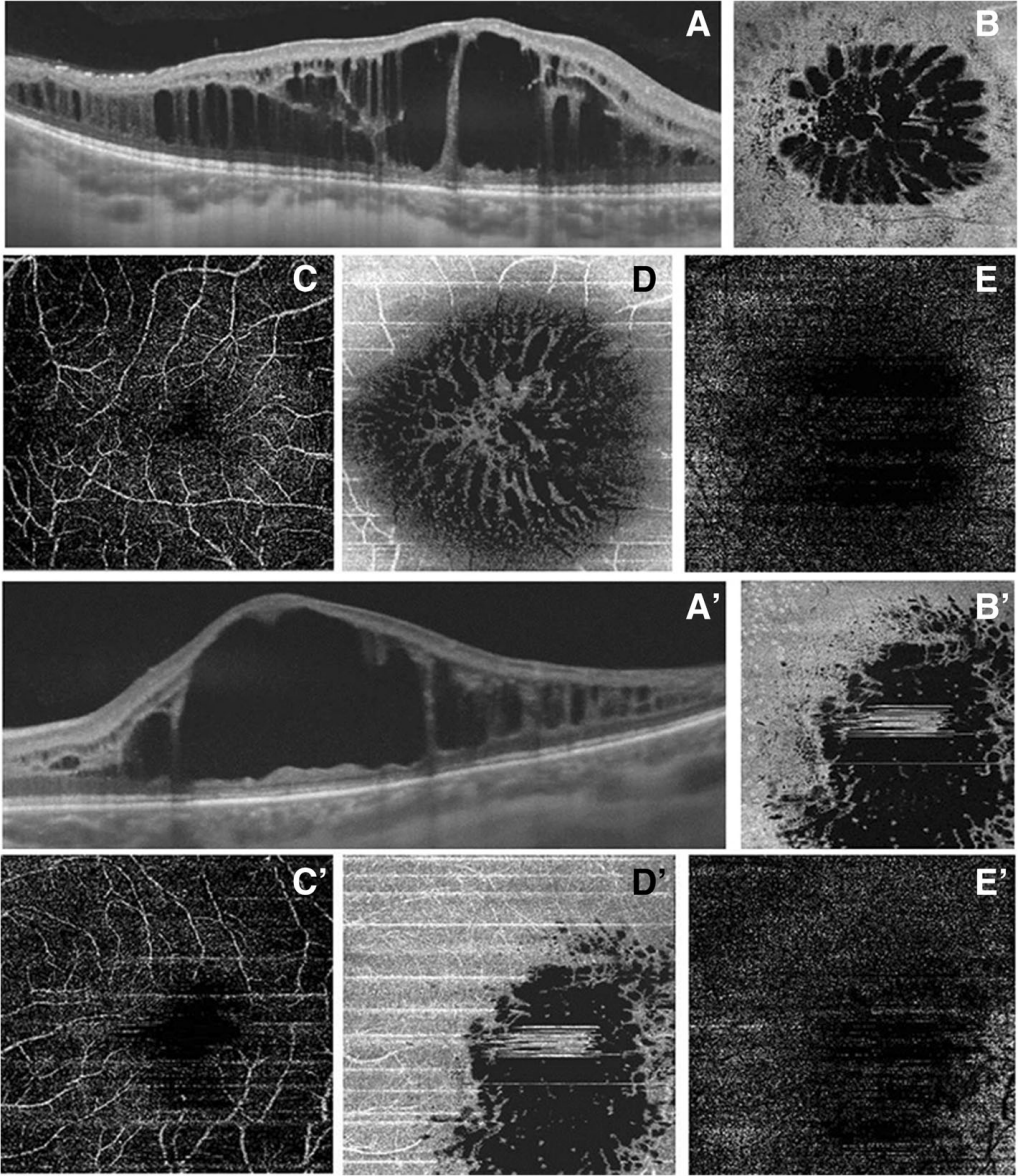
Swept source optical coherence tomography and optical coherence tomography angiography in right eye (**a**) and left eye (**a**’) affected by enhanced S-cone syndrome. Optical coherence tomography B scan shows an irregular retinal profile associated with the increase of macular thickness due to the presence of schisis both in the outer nuclear layer and in the inner nuclear layer. Vertical septa are observable mainly in right eye (**a-a**’). “En face” optical coherence tomography scans reveal the presence of hyporeflective spaces (**b-b’**). Optical coherence tomography angiography, performed at the level of superficial plexus, shows the preservation of circulation of this layer (**c-c′**). Images of the deep capillary plexus revealed a marked involvement of this vascular layer with absence of capillary details secondary to the sliding of vessels at the margin of the schisis (**d-d’**). Optical coherence tomography angiography, performed at the level of retinal avascular layer, shows the total absence of flow (**e-e’**)

**Fig. 4:**
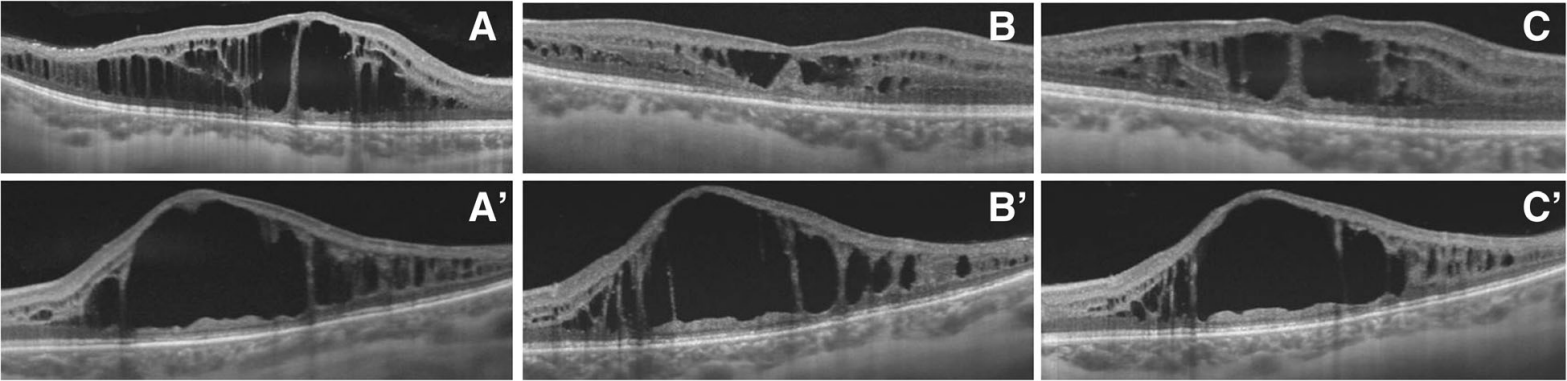
Optical coherence tomography B scan images showing the progression of macular schisis in right eye and left eye affected by enhanced S-cone syndrome. **a-a’** Horizontal structural optical coherence tomography images of right eye (*A*) and left eye (*A’*) show an increase in macular thickness due to the presence of retinal schisis involving the inner and outer nuclear layers. Hyporeflective holes are larger in the left eye, while hyper-reflective vertical septa can be better observed in the right eye. The outer plexiform layer is interrupted in both the right eye and the left eye. **b-b′** Swept source structural optical coherence tomography B scans, performed 1 month after the first examination, reveal a decrease in central macular thickness and in size of cystic-like spaces in the right eye. No differences are detectable in the left eye. **c-c′** Structural optical coherence tomography B scans, obtained 2 months after the second evaluation, point out a mild increase in retinal thickness and in size of the hyporeflective spaces in the right eye. No change can be seen in the left eye

**Fig. 5:**
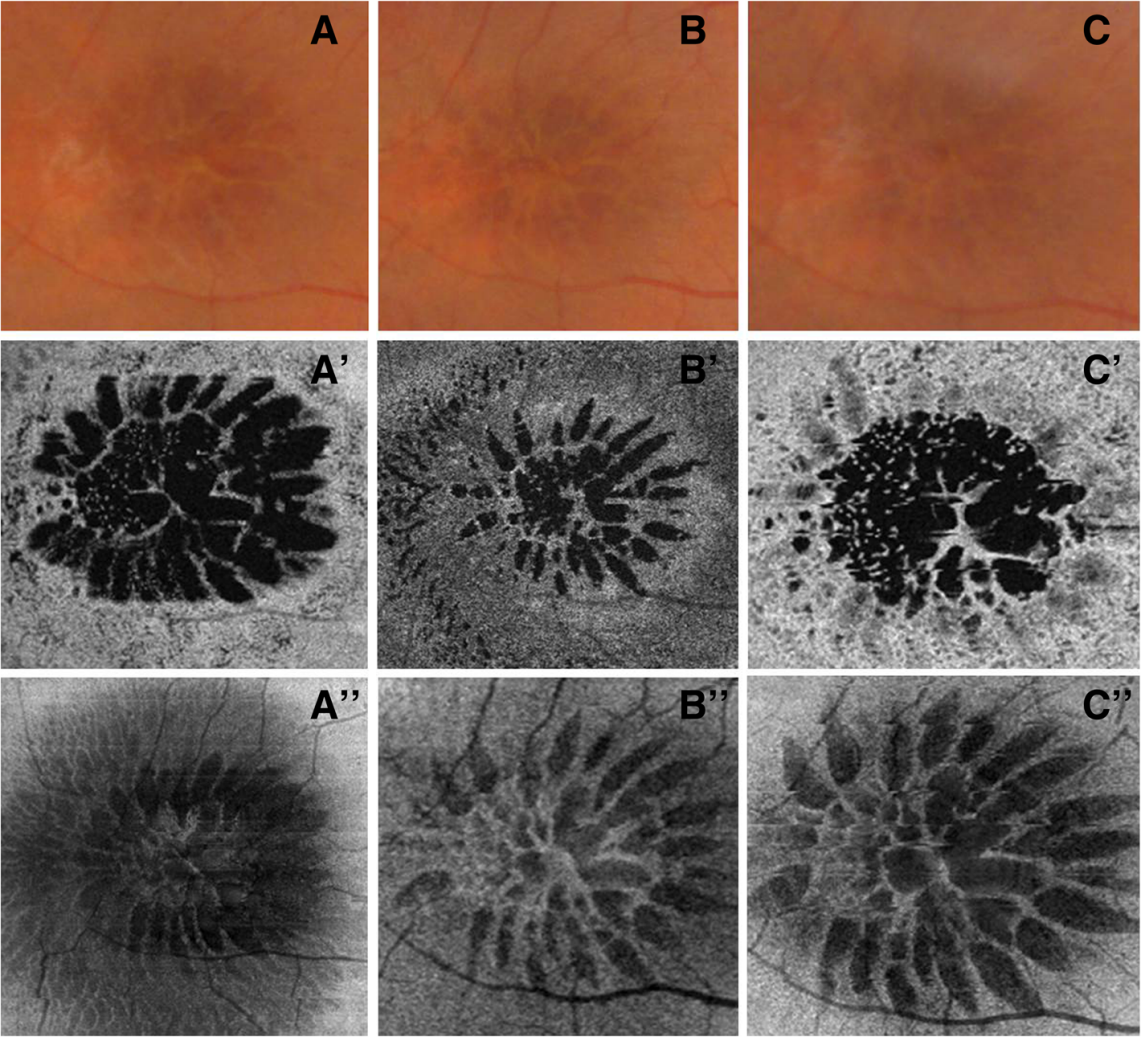
Fundus photography shows the presence of macular schisis seen as a cartwheel pattern (**a**). “En face” OCT images at the level of the inner (**a’**) and outer (**a’’**) nuclear layers point out the appearance of hyporeflective holes separated by hyperreflective septa. Cystic–like spaces are round-shaped in the inner nuclear layer, while they are elongated in the outer nuclear layer. Fundus photography (**b**) and enface OCT images (**b’**, **b’’**), performed one month after the first evaluation, reveal a reduction in number and size of the holes both in the inner (**b’**) and outer (**b’’**) nuclear layers. Fundus photography (**c**) and enface OCT scans (**c’**, **c’’**), obtained two months after the second examination, indicate a mild worsening in the number and size of the hyporeflective spaces. These features can be detected both in the inner (**c’**) and outer (**c’’**) nuclear layers

**Fig. 6:**
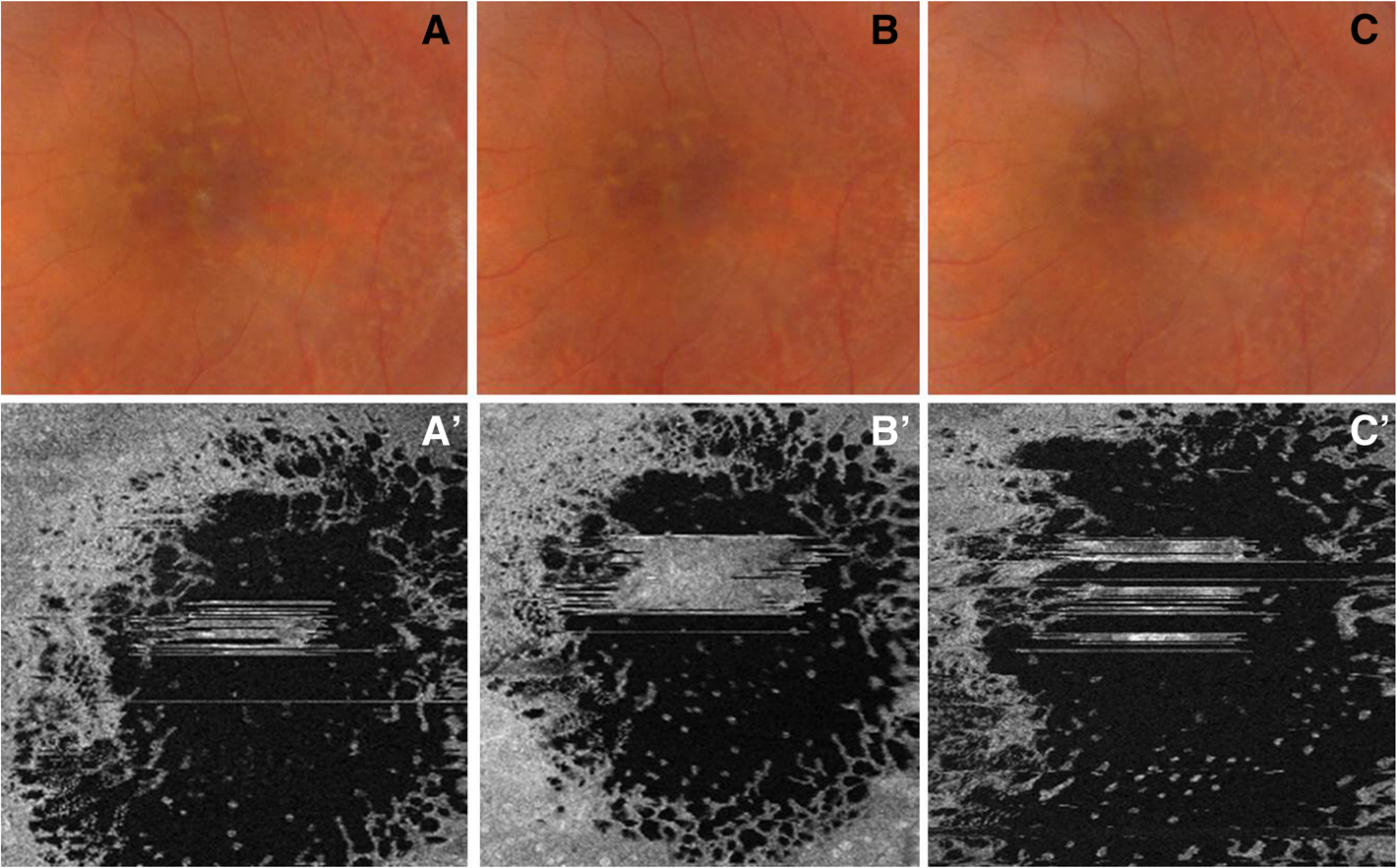
Fundus photography and “en face” optical coherence tomography images of the left eye showing the progression of macular schisis. Fundus photography (**a**) shows the presence of macular schisis seen as a cartwheel pattern. “En face” structural optical coherence tomography image **(a’**) at the level of the inner nuclear layer reveals the appearance of hyporeflective holes. **b-b′** Fundus photography and “en face” optical coherence tomography images, performed 1 month after the first evaluation, show no changes in the number and size of the holes. **c-c′** Fundus photography and “en face” optical coherence tomography scans, obtained 2 months after the second examination, reveal no difference in the number and size of the hyporeflective spaces

Macular schisis, as seen on OCT B scans (Fig. 3, a-a’) (Fig. 4, a-a’), affected both the outer and the inner nuclear layers: hyporeflective cystic-like spaces were seen in both these layers, even though they were much larger in the outer one. The outer plexiform layer appeared to be interrupted in the center of the macular region as holes in the inner and outer nuclear layers joined together. The vertical septa were observable mainly in the RE probably due to a greater disruption of retinal tissue in the LE. Hyporeflective holes separated by hyper-reflective partitions could also be visualized in the macular region on “en face” OCT images (Fig. 3, b-b’) (Fig. 5, a’-a″) (Fig. 6, a’) performed at the level of the inner and outer nuclear layers. These spaces had different shapes: they were round-shaped in the inner nuclear layers, oval in the outer one.

At the same time an OCT-A was performed using the same device and it enabled the visualization of the vascular involvement. Superficial and deep vascular network can be separately evaluated by OCT-A. OCT-A scans of the superficial plexus showed the insignificant involvement of circulation of this layer (Fig. 3, c-c′) in both eyes, while the deep plexus revealed a marked remodeling of this vascular layer. In fact, there was an absence of capillary details secondary to the sliding of vessels at the margin of the schisis (Fig. 3, d-d’). The choriocapillaris vascular network did not seem to be affected by any abnormalities.

Changes in retinal structure were monitored by performing swept source OCT 1 month and 3 months after the first evaluation: 1 month later, a decrease in retinal thickness and in size of the hyporeflective cavities were noticed on macular OCT B scans carried out on her RE (Fig. 4, b); these features were consistent with the aspect observed on “en face” scans (Fig. 5, b’-b″) which showed a reduction in number and size of the cystic-like spaces. Three months after the first examination, a mild deterioration was detected (Fig. 4, c) (Fig. 5, c′-c″). A general improvement in retinal structure was congruent with visual acuity increase from 65 letters ETDRS to 80 letters; by contrast, no difference was noticed on horizontal scans (Fig. 4, a’-b′-c′) and on “en face” OCT images (Fig. 6, a’-b’-c’) performed on her LE, while visual acuity improved from 40 letters ETDRS to only 50 letters.

Genetic analysis showed two variants in *NR2E3* (NM_014249.3): a missense variation c.1118T>C, which leads to substitution of leucine with proline in amino acid position 373, and c.349+5G>C, which involves a gene sequence near a splicing site.

## Discussion

Enhanced S-cone dystrophy is a rare condition which involves the macula with severe visual loss and could be associated with retinal schisis. Here we report a case of ESCS associated with variations in *NR2E3*, a characteristic pattern of ERG, and new findings on OCT-A.

Genetic testing revealed that the proband was heterozygous for the p.(Leu373Pro) and the c.349+5G>C variants in the *NR2E3* gene. Of interest, neither variant has been linked to any form of retinopathy. The missense variant is not listed in any of the public databases questioned, such as the Exome Aggregation Consortium (ExAC) database (exac.broadinstitute.org/), 1000 Genomes (http://www.internationalgenome.org/), TOPMed (https://www.nhlbiwgs.org/), and the Exome Variant Server (EVS; http://evs.gs.washington.edu/EVS/) database. It is predicted to be deleterious and probably damaging by SIFT (http://provean.jcvi.org/genome_submit_2.php) and PolyPhen-2 (http://genetics.bwh.harvard.edu/pph2/) *in silico* prediction software, respectively. The variant lies in the poly-leucine segment of the ligand-binding domain (LBD) of the protein. Tan *et al.* [19] suggested that NR2E3 auto-represses itself by taking the dimeric conformation required for its repressor functions. Since the S-cone is the default state of a generic photoreceptor precursor [20], and NR2E3 repressor function has been proven to be necessary to consolidate the rod fate of rod precursor cells, variations affecting this function therefore result in abnormal cell-fate determination, leading to excess S-cones at the expense of other photoreceptor subtypes [9]. Tan *et al*. [19] demonstrated that leucine in position 372 and leucine in position 375 in the poly-leucine segment are important for dimer formation and variation in this residue affects NR2E3 repressor activity. The variant p.(Leu373Pro) could influence the dimerization, if not in a direct way, perhaps by disturbing the interaction of the amino acids involved in this process, thus explaining the ESCS of our patient. However, this can only be demonstrated by functional studies.

The on-line tool Human Splicing Finder (http://www.umd.be/HSF3/) predicts that c.349+5G>C (rs760771835; MAF:0.00002% in TOPMed) breaks a wild-type donor site in intron 3, probably affecting splicing; however, this software is unable to predict whether the protein will be subject to exon skipping or intron retention.

A family segregation study revealed that the variations were *in trans* configuration in the proband; in fact, her mother was heterozygous for c.349+5G>C while her father carried the p.(Leu373Pro). The proband’s relatives were healthy, thus confirming the autosomal recessive transmission of the disease.

In our experience, swept source OCT imaging has represented an effective way to investigate the involvement of each retinal layer (using “en face” images) and to monitor structural retinal changes over time (using both horizontal and “en face” scans).

In this case of retinal dystrophy, the “en face” images allowed suitable observation of the schisis both involving the inner and outer nuclear layer. The hyporeflective spaces had a rounded aspect in the inner nuclear layer, whereas in the outer layer the cavities were elongated with a stellate pattern; this peculiar feature is due to the Müller cells distribution around the fovea as a “Z-shaped” course in the axial plane, running obliquely in the outer plexiform layer and in the Henle fiber layer, and vertically in the inner layers [21].

Similarly, the use of OCT-A allowed the visualization of the degree of vascular involvement in ESCS.

In general, the importance of OCT-A in separately investigating the retinal superficial and deep vascular plexuses and the choriocapillaris has been underlined [22]. In our observation, the superficial network seems to be only partially involved while the deep network is mainly compromised. In particular, the typical features of tiny fans, pathognomonic of the deep network, were not observable.

## Conclusion

This report was intended to describe details of “en face” swept source OCT and OCT-A in a case of ESCS associated with bilateral retinal schisis, which, to the best of our knowledge, has never been reported previously. We believe “en face” OCT and OCT-A can provide useful, non-invasive, and safe tools of investigation to detect structural changes in the retinal morphology and vascular structure related to the ESCS.

The clinical novelty of this case report is increased by the genetic findings of two variations in the *NR2E3* gene here described for the first time in association with ESCS.

## Abbreviations

BCVA: Best corrected visual acuity
ERG: Electroretinography
ESCS: Enhanced S-cone syndrome
ETDRS: Early Treatment Diabetic Retinopathy Study
EVS: Exome Variant Server
ExAC: Exome Aggregation Consortium
ISCEV: International Society for Clinical Electrophysiology of Vision
LBD: Ligand-binding domain
LE: Left eye
ML: Medium Long-wavelenght sensitive
*NR2E3*: Nuclear receptor subfamily 2 group E member 3
OCT: Optical coherence tomography
OCT-A: Optical coherence tomography angiography
RE: Right eye
